# Supplementary material for: Local halide heterogeneity drives surface wrinkling in mixed-halide wide-bandgap perovskites
Source: Nat Commun. 2025 Feb 25;16:1967. doi: 10.1038/s41467-025-57010-6 (PMC11861982; doi:10.1038/s41467-025-57010-6)
Supplement: Supplementary file 1 — Supplementary Information [file 41467_2025_57010_MOESM1_ESM.pdf]

Supplementary Information

Local halide heterogeneity drives surface wrinkling in mixed-halide wide-bandgap perovskites

Kunal Datta, Simone C. W. van Laar, Margherita Taddei, Juanita Hidalgo, Tim Kodalle, Guus J. W. Aalbers, Barry Lai, Ruipeng Li, Nobumichi Tamura, Jordi T. W. Frencken, Simon V. Quiroz Monnens, Robert J. E. Westbrook, Daniel J. Graham, Carolin M. Sutter-Fella, Juan-Pablo Correa-Baena, David S. Ginger, Martijn M. Wienk, and René A. J. Janssen

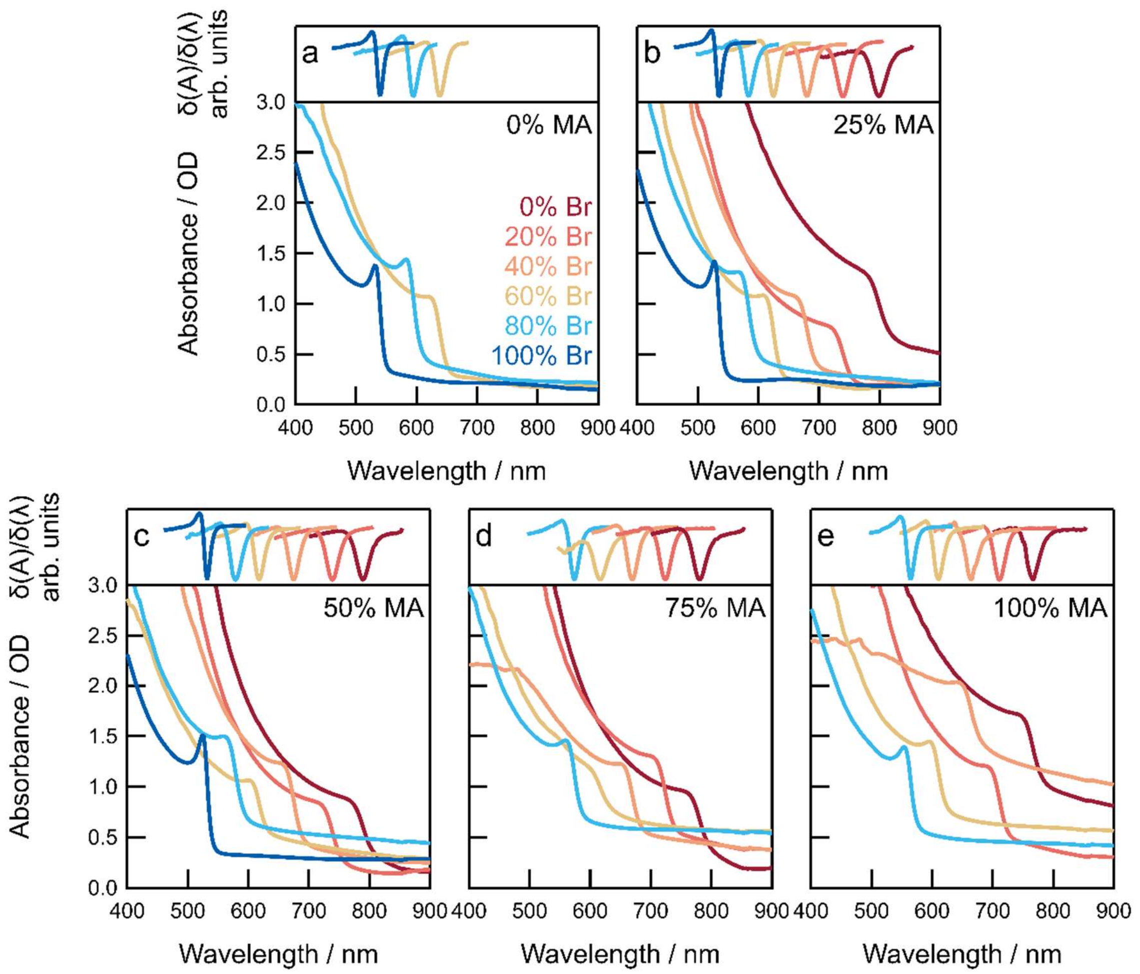

Supplementary Fig. 1 | UV-vis-NIR spectra of $(FA_{1-x}MA_x)Pb(I_{1-y}Br_y)_3$ perovskite thin films with varied MA (x) and Br contents (y). **a** $\{x\} = 0$. **b** $\{x\} = 0.25$. **c** $\{x\} = 0.50$. **d** $\{x\} = 0.75$. **e** $\{x\} = 1.0$. Top panels represent the first derivative of the absorption spectrum from where the minima were used to identify the optical bandgap.

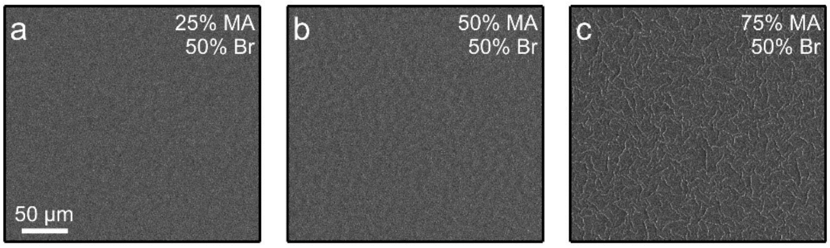

Supplementary Fig. 2 | Surface SEM images of $(FA_{1-x}MA_x)Pb(I_{1-y}Br_y)_3$ perovskite films with 50% Br content and different MA contents. **a** $x = 0.25$. **b**. $x = 0.50$. **c**. $x = 0.75$.

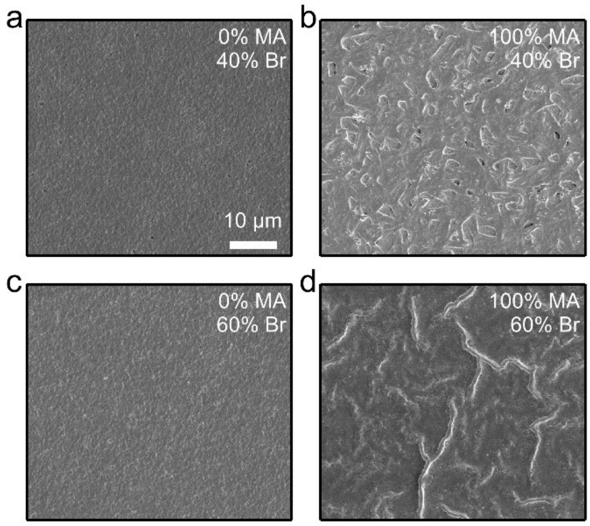

Supplementary Fig. 3 | Surface SEM images of $(FA_{1-x}MA_x)Pb(I_{1-y}Br_y)_3$ perovskite thin films for different compositions. **a** {*x*/*y*} = 0|0.40. **b**. {*x*/*y*} = 1.0⁝0.40. **c**. {*x*/*y*} = 0|0.60. **d**. {*x*/*y*} = 1.0⁝0.60.

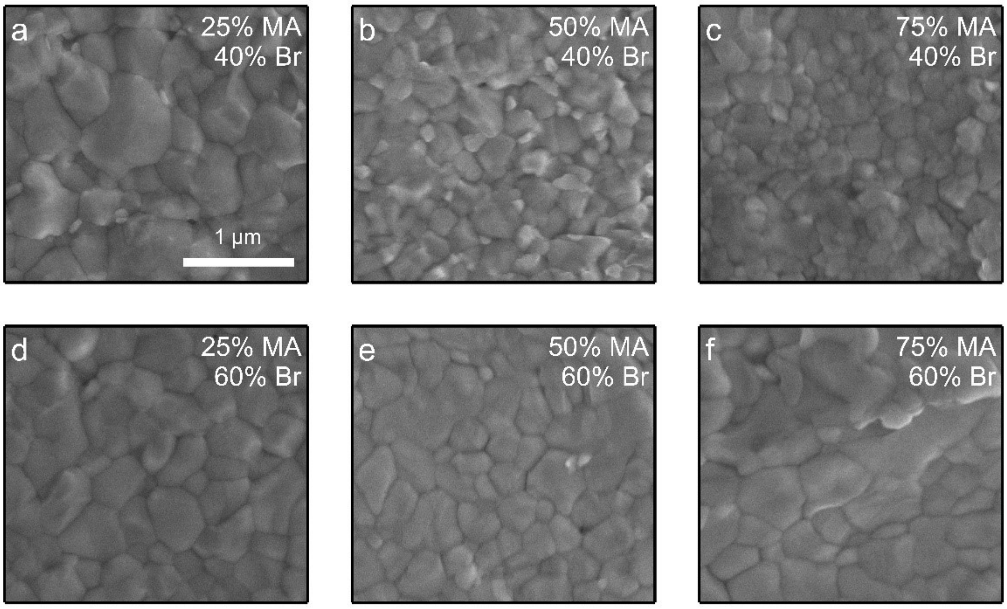

Supplementary Fig. 4 | High magnification surface SEM images of $(FA_{1-x}MA_x)Pb(I_{1-y}Br_y)_3$ perovskite films with different compositions. **a** {*x*/*y*} = 0.25|0.40. **b** {*x*/*y*} = 0.50|0.40. **c** {*x*/*y*} = 0.75⁝0.40. **d** {*x*/*y*} = 0.25¦0.60. **e** {*x*/*y*} = 0.50⁝0.60. **f** {*x*/*y*} = 0.75⁝0.60.

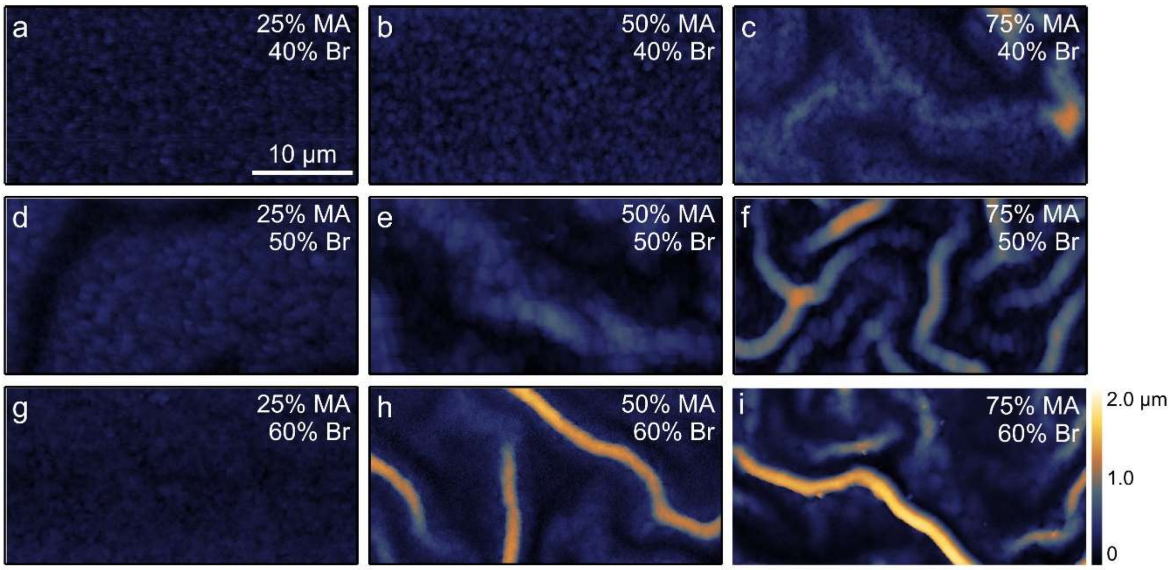

Supplementary Fig. 5 | Atomic force microscopy height profiles of $(FA_{1-x}MA_x)Pb(I_{1-y}Br_y)_3$ perovskite thin films. **a** $\{x/y\}$ = 0.25|0.40. **b** $\{x/y\}$ = 0.50|0.40. **c** $\{x/y\}$ = 0.75⁝0.40. **d** $\{x/y\}$ = 0.25|0.50. **e** $\{x/y\}$ = 0.50¦0.50. **f** $\{x/y\}$ = 0.75⁝0.50. **g** $\{x/y\}$ = 0.25¦0.60. **h** $\{x/y\}$ = 0.50⁝0.60. **i** $\{x/y\}$ = 0.75⁝0.60. Scale bars are 10 µm. Height range is from 0 – 2.0 µm.

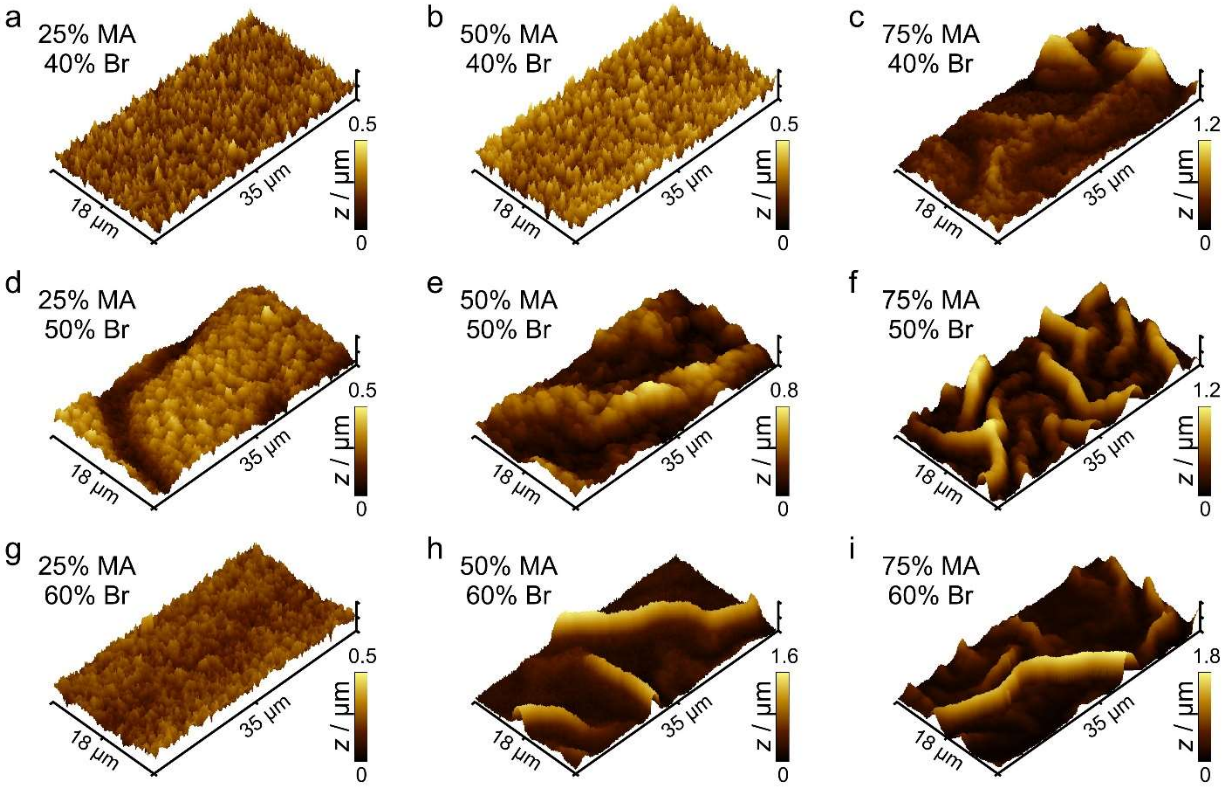

Supplementary Fig. 6 | Three-dimensional atomic force microscopy height profiles of $(FA_{1-x}MA_x)Pb(I_{1-y}Br_y)_3$ perovskite thin films. **a** {*x*/*y*} = 0.25|0.40. **b** {*x*/*y*} = 0.50|0.40. **c** {*x*/*y*} = 0.75⁞0.40. **d** {*x*/*y*} = 0.25|0.50. **e** {*x*/*y*} = 0.50¦0.50. **f** {*x*/*y*} = 0.75⁞0.50. **g** {*x*/*y*} = 0.25¦0.60. **h** {*x*/*y*} = 0.50⁞0.60. **i** {*x*/*y*} = 0.75⁞0.60.

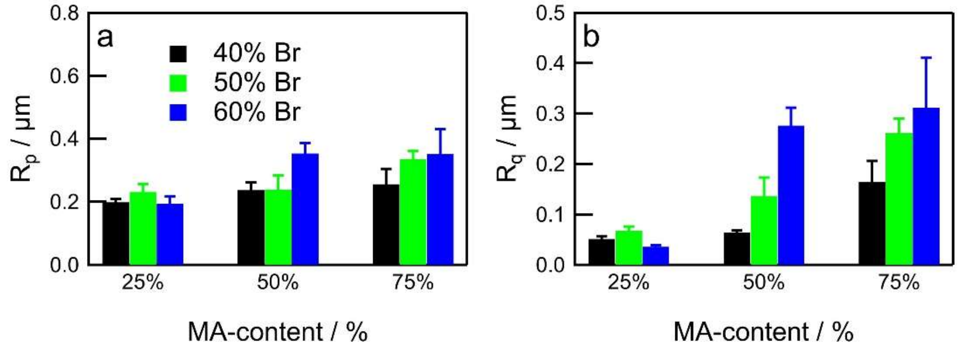

Supplementary Fig. 7 | Film roughness as a function of $(FA_{1-x}MA_x)Pb(I_{1-y}Br_y)_3$ perovskite composition. **a** Average maximum peak profile height (R_p). **b** Root mean square average roughness (R_q).

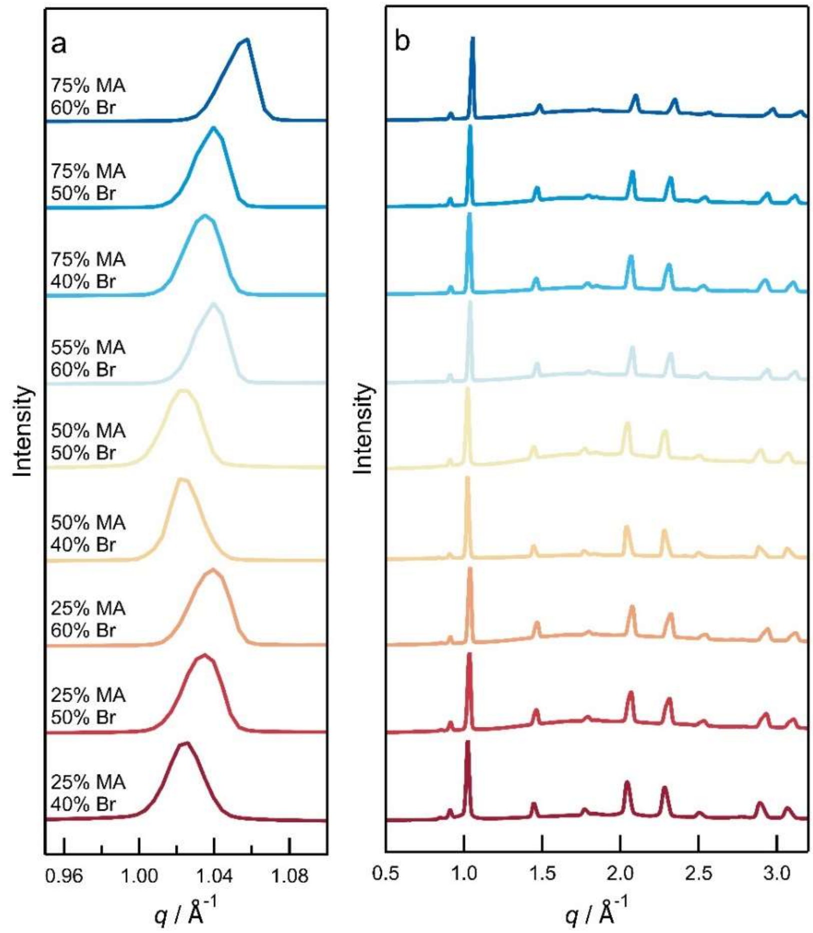

Supplementary Fig. 8 | Circular integration profile acquired from GIWAXS patterns of $(FA_{1-x}MA_x)Pb(I_{1-y}Br_y)_3$ perovskites. Data are shown for compositions: $\{x/y\}$ = 0.25|0.40, 0.25|0.50, 0.25¦0.60, 0.50|0.40, 0.50¦0.50, 0.50⁝0.60, 0.75⁝0.40, 0.75⁝0.50, and 0.75⁝0.60. **a** Circular averages centered on the (100) peak from the perovskite structure. **b** The circular average across a broad range of scattering vectors.

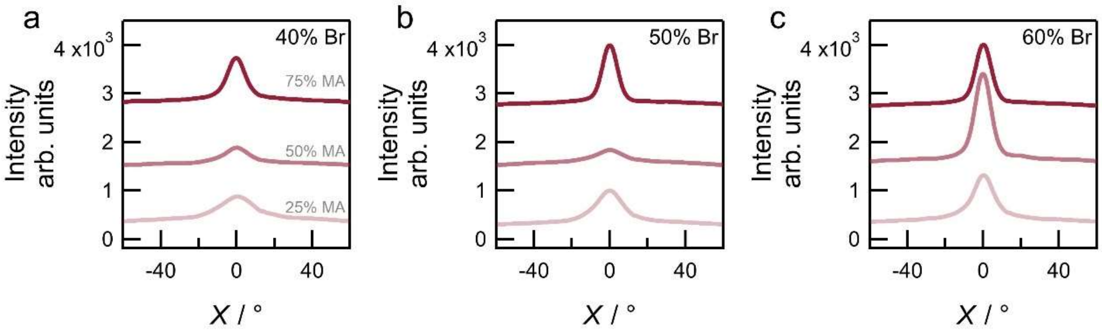

Supplementary Fig. 9 | Orientation of PbI_2 from GIWAXS patterns of $(FA_{1-x}MA_x)Pb(I_{1-y}Br_y)_3$ perovskites. Azimuthal intensity profiles of the Debye-Scherrer ring associated to PbI_2 (q = 0.9 Å^{-1}) as a function of χ angle from GIWAXS for perovskite compositions with Br content: **a** 40%, **b** 50%, and **c** 60%. The MA content is varied between 25%, 50%, and 75%.

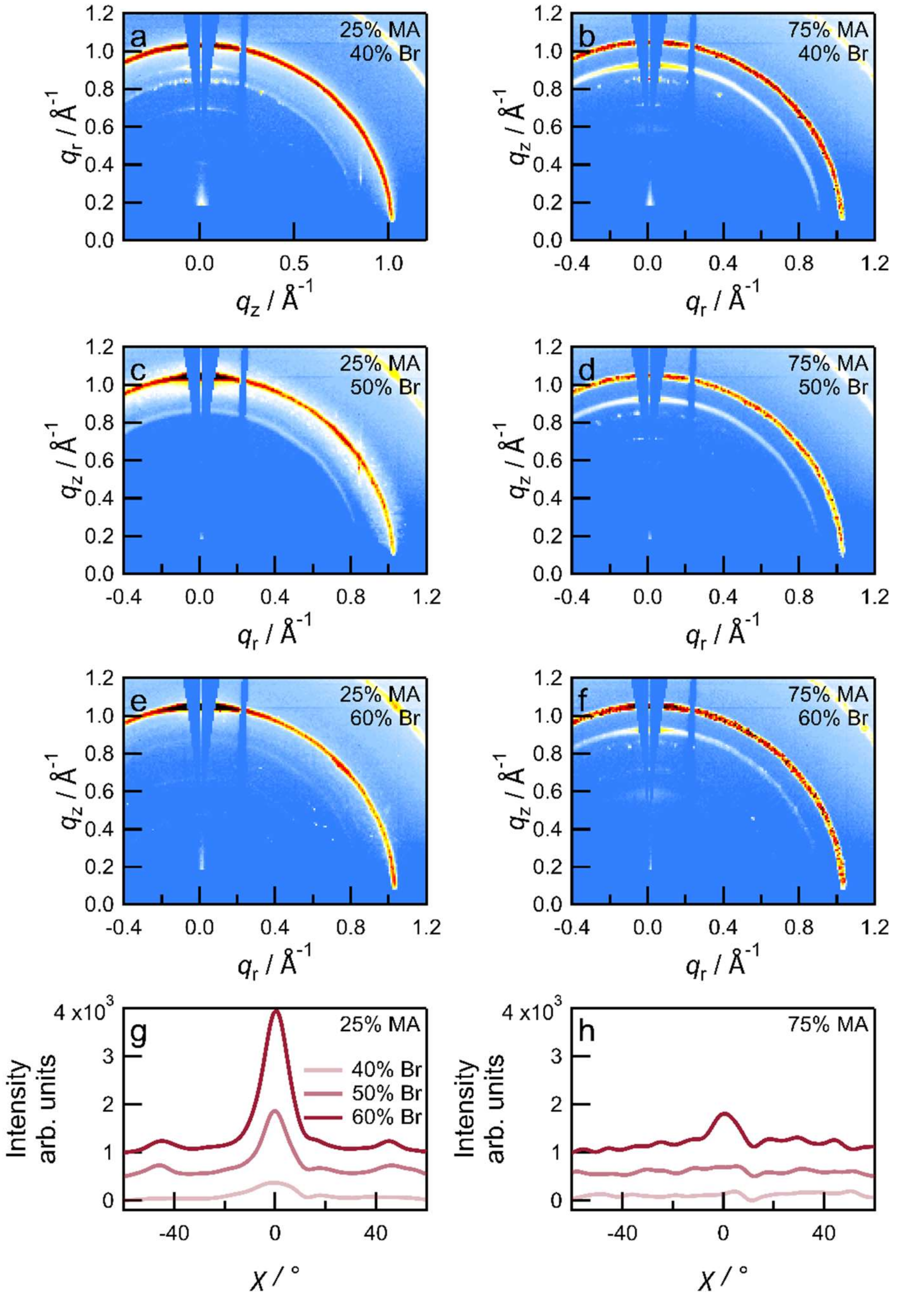

Supplementary Fig. 10 | GIWAXS patterns of $(FA_{1-x}MA_x)Pb(I_{1-y}Br_y)_3$ perovskite films prepared with the interdiffusion method. **a** $\{x/y\}$ = 0.25|0.40. **b** $\{x/y\}$ = 0.75|0.40. **c** $\{x/y\}$ = 0.25|0.50. **d** $\{x/y\}$ = 0.75|0.50. **e** $\{x/y\}$ = 0.25|0.60. **f** $\{x/y\}$ = 0.75|0.60. Azimuthal intensity profiles of the main Debye-Scherrer ring (100) as a function of χ angle from GIWAXS for perovskite compositions with varying MA- and Br-contents. **g** 25% MA. **h** 75% MA. The data in panels **g** and **h** have been vertically offset for clarity.

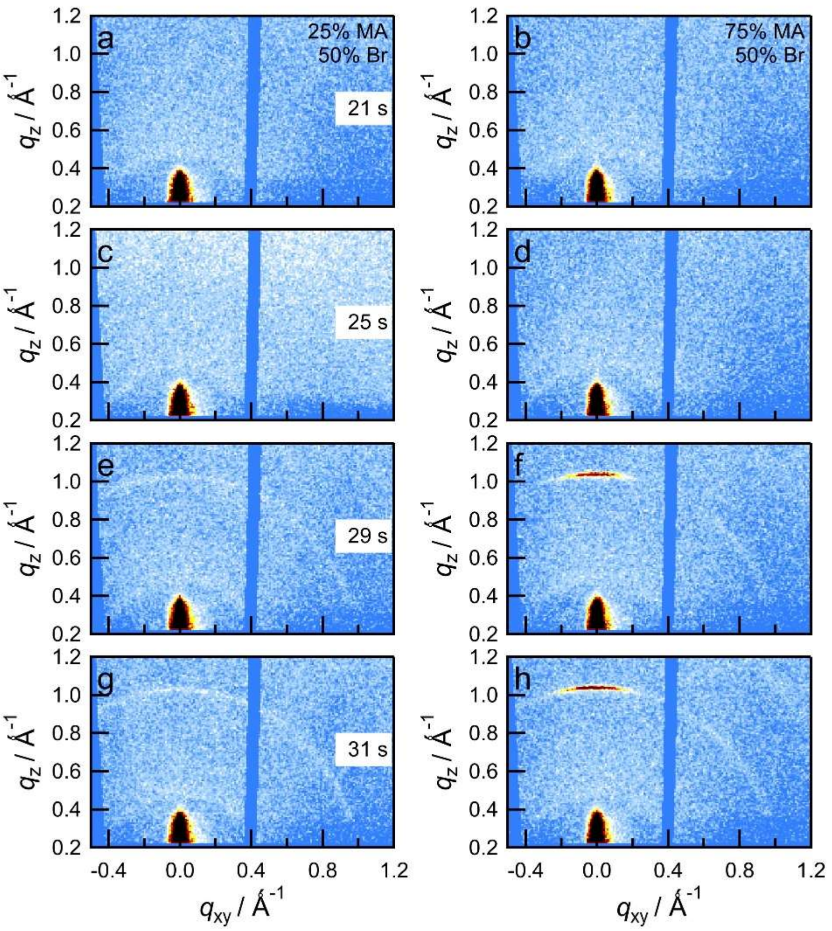

Supplementary Fig. 11 | In situ GIWAXS patterns acquired at different times during spin-coating of $(FA_{1-x}MA_x)Pb(I_{1-y}Br_y)_3$ perovskite films. **a**, **c**, **e**, and **g** {*x*/*y*} = 0.25|0.50. **b**, **d**, **f**, and **h** 0.75⁝0.50. The anti-solvent is applied after 25 s (panels **c** and **d**).

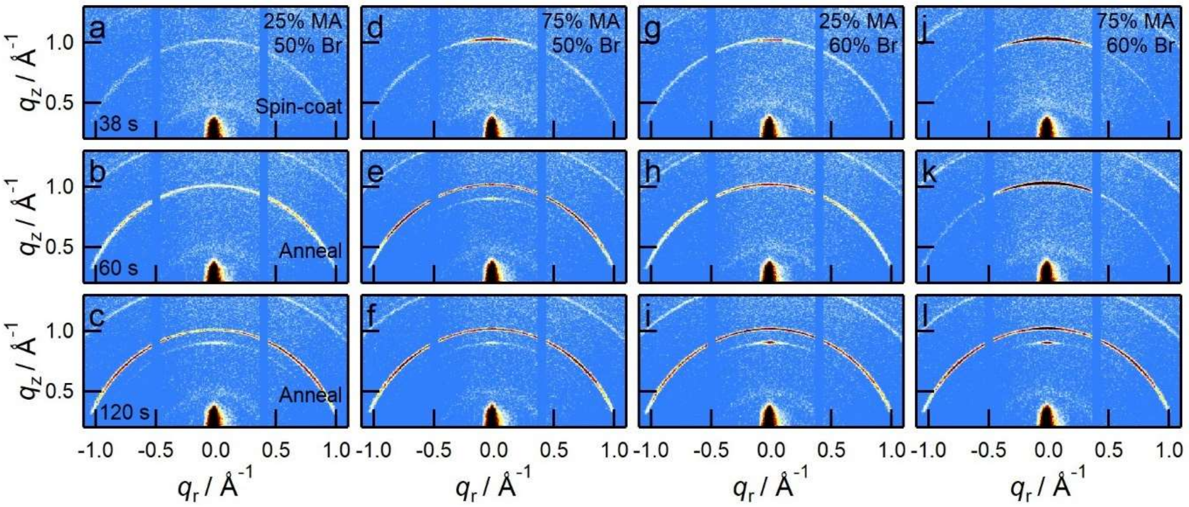

Supplementary Fig. 12 | In situ GIWAXS patterns acquired at different processing times for $(FA_{1-x}MA_x)Pb(I_{1-y}Br_y)_3$ perovskite films. **a – c** {*x*/*y*} = 0.25|0.50, **d – f** {*x*/*y*} = 0.75⁝0.50, **g – i** {*x*/*y*} = 0.25¦0.60, **j – l** {*x*/*y*} = 0.75⁝0.60.

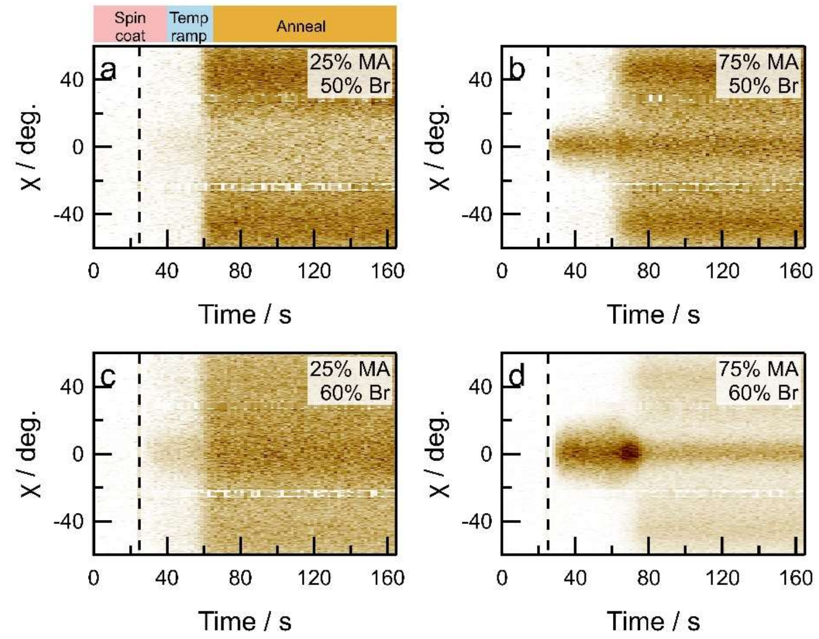

Supplementary Fig. 13 | 2D maps of azimuthal integration profiles of (100) ring as a function of time during ($FA_{1-x}MA_x$)Pb($I_{1-y}Br_y$)$_3$ perovskite film processing. **a** {*x*/*y*} = 0.25|0.50. **b** {*x*/*y*} = 0.75⁝0.50. **c** {*x*/*y*} = 0.25¦0.60. **d** {*x*/*y*} = 0.75⁝0.60. The dashed vertical line represents the antisolvent drop.

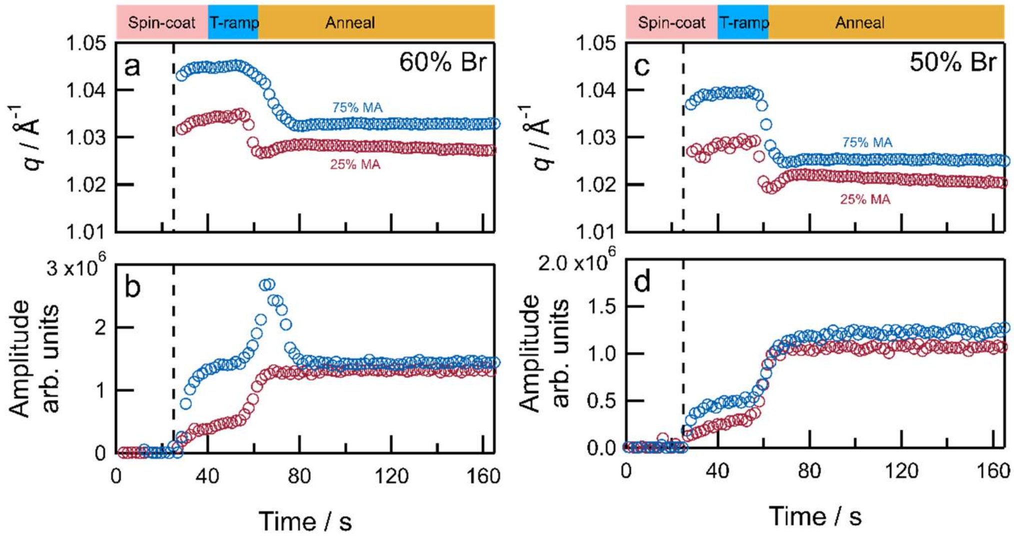

Supplementary Fig. 14 | In situ GIWAXS during ($FA_{1-x}MA_x$)Pb($I_{1-y}Br_y$)$_3$ perovskite film processing. **a** *q*-value of the (100) peak as a function of crystallization time for perovskite films with compositions {*x*/*y*} = 0.25¦0.60 and 0.75⁝0.60. **b** Corresponding intensity of the (100) peak as a function of time. The dashed vertical line marks the antisolvent drop. **c** *q*-value of the (100) peak as a function of crystallization time for perovskite films with compositions {*x*/*y*} = 0.25|0.50 and 0.75⁝0.50. **d** Corresponding intensity of the (100) peak as a function of time. The dashed vertical line marks the antisolvent drop.

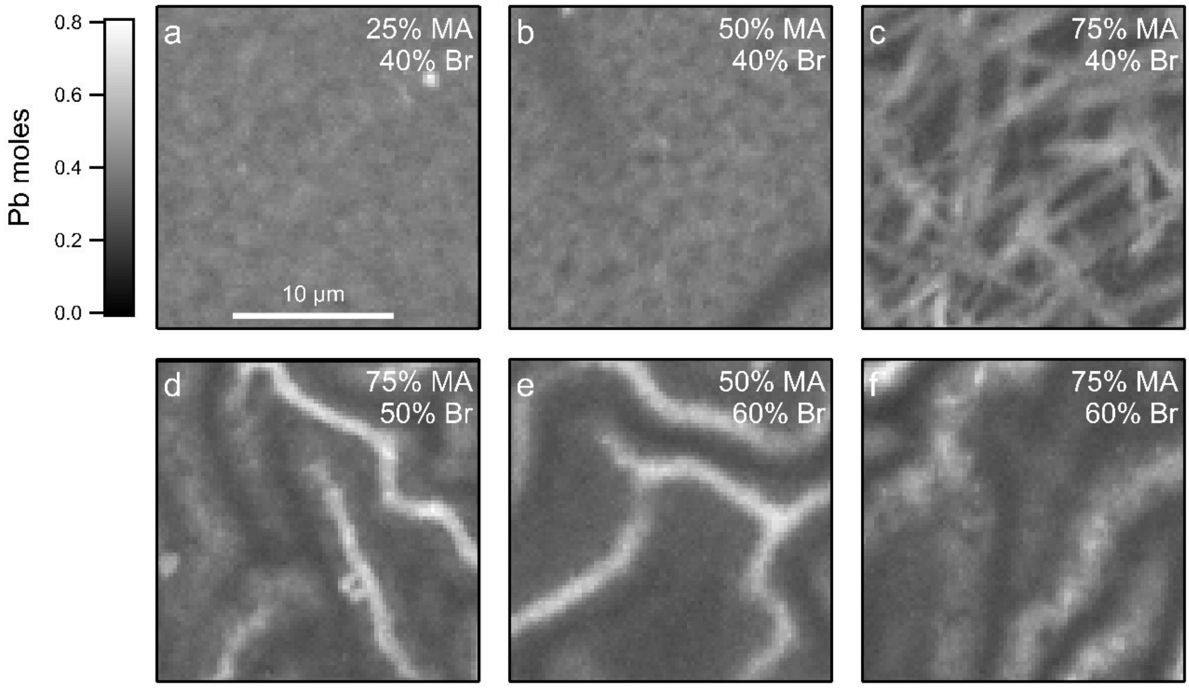

Supplementary Fig. 15 | Nano-XRF maps of Pb-content in $(FA_{1-x}MA_x)Pb(I_{1-y}Br_y)_3$ perovskite films with different compositions. **a** {*x*/*y*} = 0.25|0.40. **b** {*x*/*y*} = 0.50|0.40. **c** {*x*/*y*} = 0.75⁝0.40. **d** {*x*/*y*} = 0.75⁝0.50. **e** {*x*/*y*} = 0.50⁝0.60. **f** {*x*/*y*} = 0.75⁝0.60.

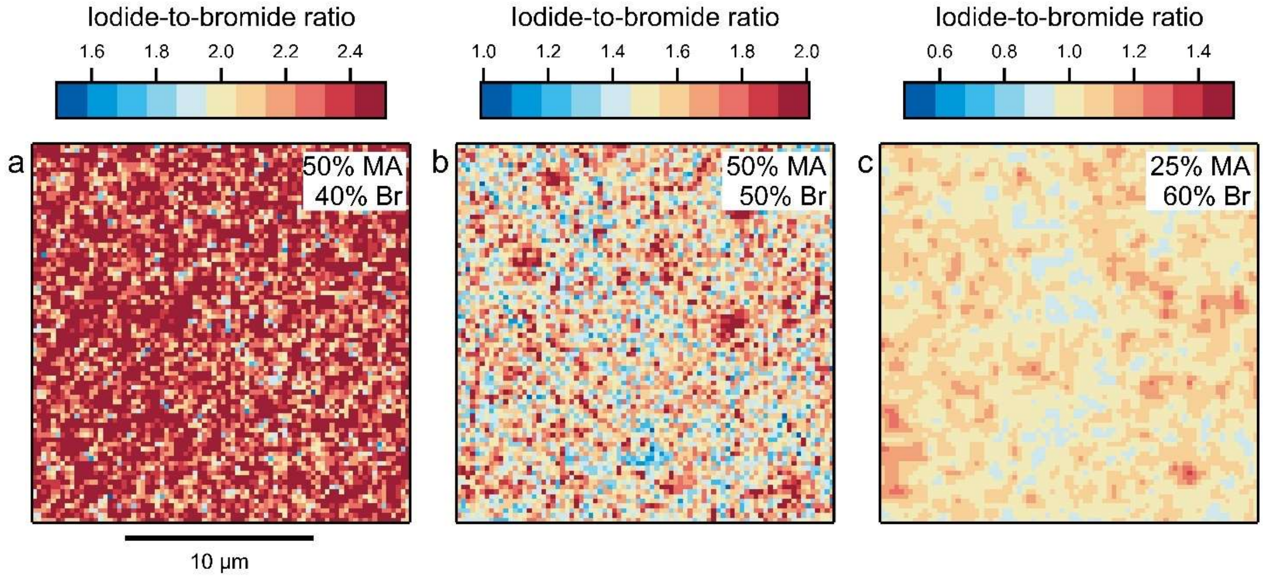

Supplementary Fig. 16 | Iodide-to-bromide ratios acquired using I and Br elemental nano-XRF mapping in $(FA_{1-x}MA_x)Pb(I_{1-y}Br_y)_3$ perovskite films with different compositions. **a** {*x*/*y*} = 0.50|0.40. **b** {*x*/*y*} = 0.50¦0.50. **c** {*x*/*y*} = 0.25¦0.60.

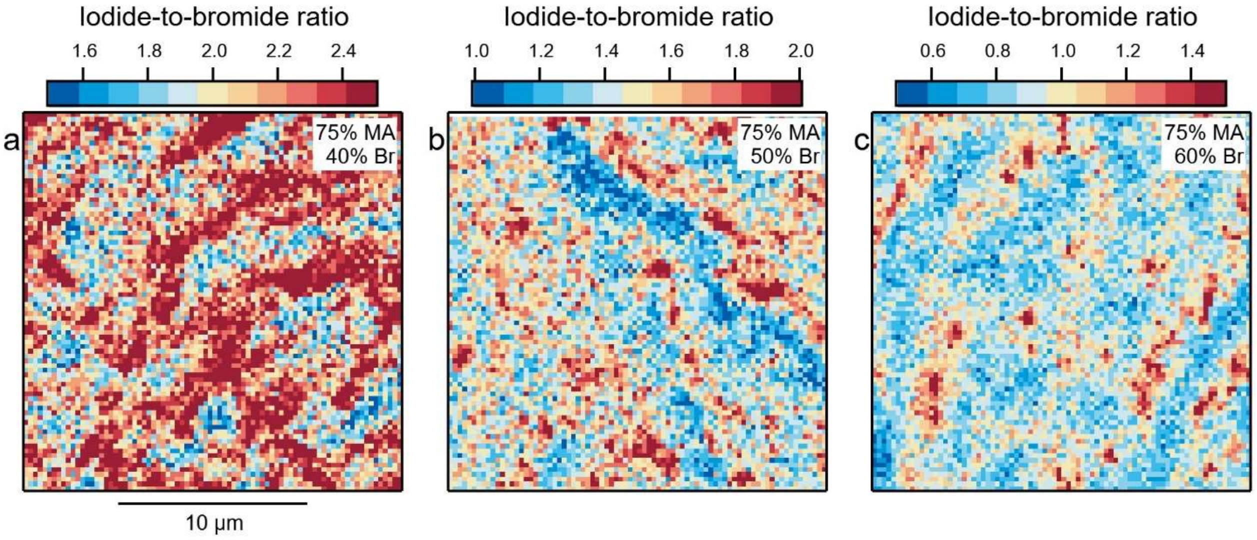

Supplementary Fig. 17 | Iodide-to-bromide ratios acquired using I and Br elemental nano-XRF mapping in $(FA_{1-x}MA_x)Pb(I_{1-y}Br_y)_3$ perovskite films with different compositions. a {*x*/*y*} = 0.75⁝0.40. **b** {*x*/*y*} = 0.75⁝0.50. **c** {*x*/*y*} = 0.75⁝0.60.

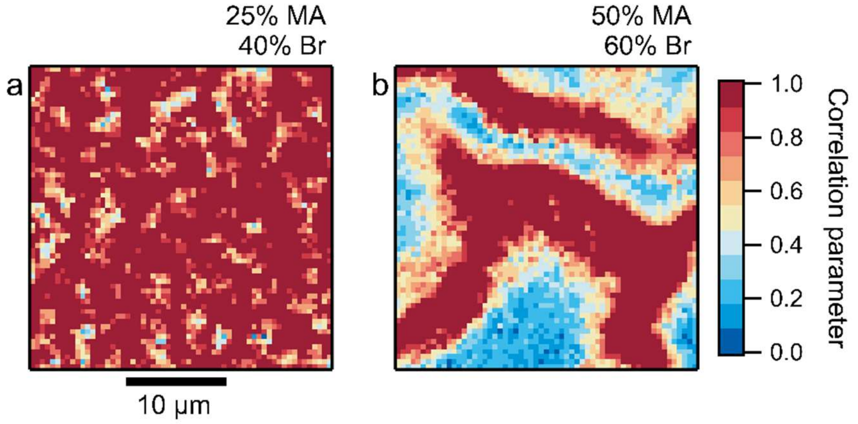

Supplementary Fig. 18 | Correlation between iodide-to-bromide ratio and lead content in $(FA_{1-x}MA_x)Pb(I_{1-y}Br_y)_3$ perovskite films. a {*x*/*y*} = 0.25|0.40. **b** {*x*/*y*} = 0.50⁝0.60. In order to calculate the correlation factor, two-dimensional maps of iodide-to-bromide content and Pb-content were normalized and the ratio between the two were taken. The maps show that while for a smooth film ({*x*/*y*} = 0.25|0.40), a positive correlation (correlation factor ~ 1) appears almost homogeneously across the surface, in a rough film ({*x*/*y*} = 0.50⁝0.60), such a positive relationship only appears at wrinkled regions.

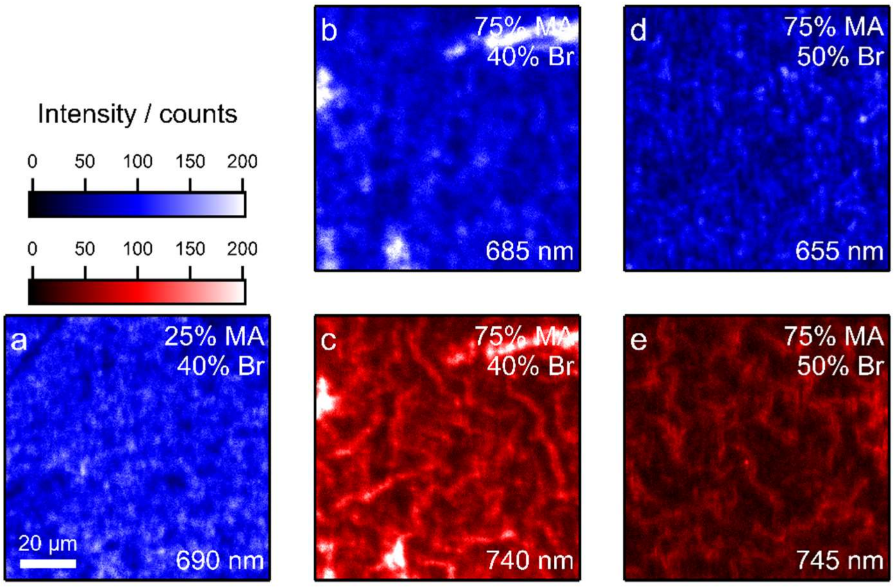

Supplementary Fig. 19 | Photoluminescence intensity maps of pristine $(FA_{1-x}MA_x)Pb(I_{1-y}Br_y)_3$ perovskite films. **a** $\{x/y\}$ = 0.25|0.40, **b, c** $\{x/y\}$ = 0.75¦0.40. **d, e** $\{x/y\}$ = 0.75¦0.50. Each panel indicates the wavelength at which the emission map is measured.

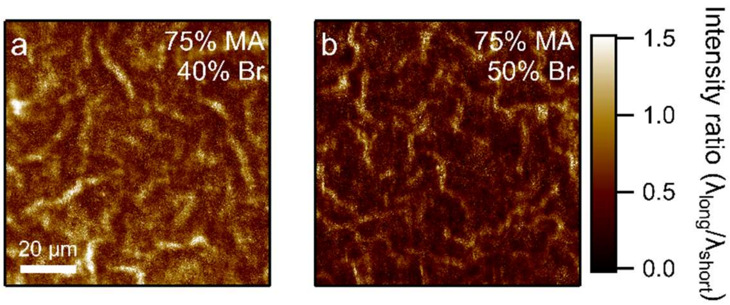

Supplementary Fig. 20 | Photoluminescence maps of pristine $(FA_{1-x}MA_x)Pb(I_{1-y}Br_y)_3$ perovskite films. PL intensity ratios of long- and short-wavelength emissions for perovskite films with compositions **a** $\{x/y\}$ = 0.75¦0.40 (λ_{short} = 685 nm, λ_{long} = 740 nm), **b** $\{x/y\}$ = 0.75¦0.50 (λ_{short} = 655 nm, λ_{long} 745 nm).

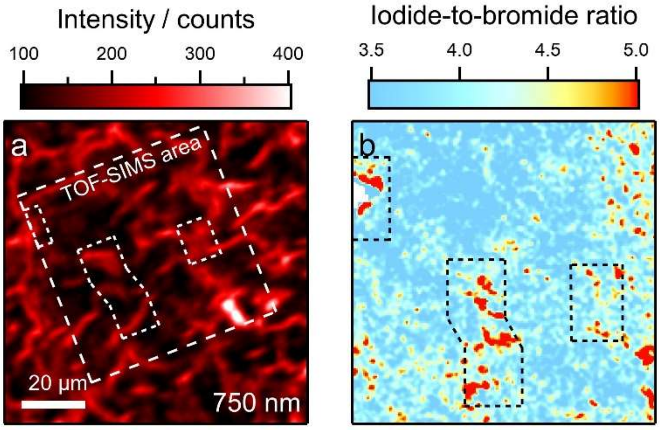

Supplementary Fig. 21 | PL and TOF-SIMS map of pristine $(FA_{1-x}MA_x)Pb(I_{1-y}Br_y)_3$ perovskite film. a PL intensity map (750 nm) for perovskite composition {*x*/*y*} = 0.75⁝0.50. Dashed square represents sample area was used for TOF-SIMS measurement. **b** Corresponding TOF-SIMS map of I^-/Br^- ratio.

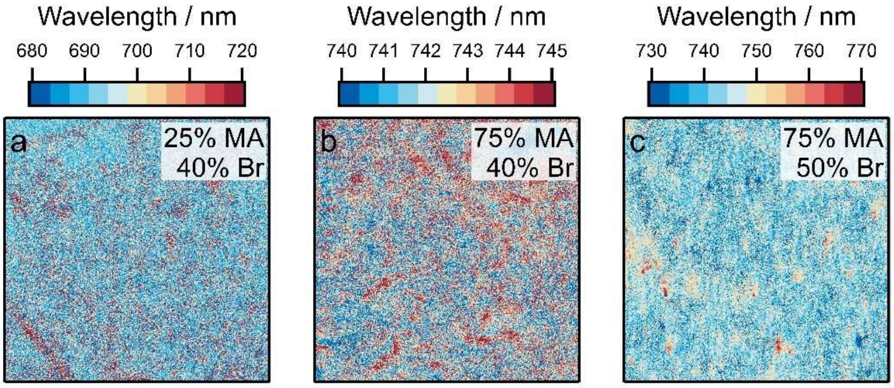

Supplementary Fig. 22 | Photoluminescence peak maps after continuous illumination for $(FA_{1-x}MA_x)Pb(I_{1-y}Br_y)_3$ perovskite films with different compositions. **a** {*x*/*y*} = 0.25|0.40. **b** {*x*/*y*} = 0.75⁝0.40. **c** {*x*/*y*} = 0.75⁝0.50. Note the different color scales in the three panels.

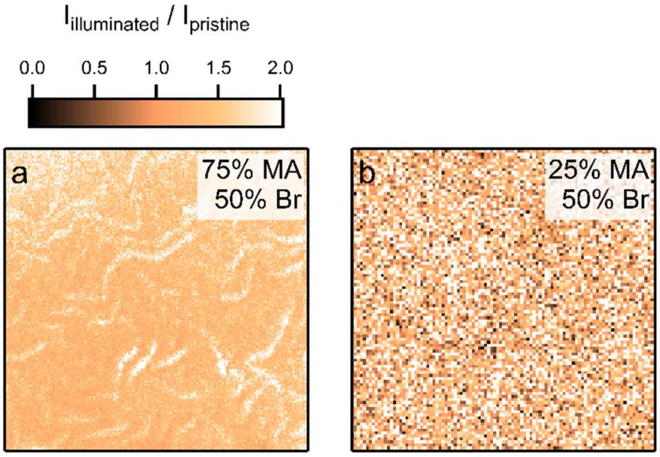

Supplementary Fig. 23 | Ratio of surface iodide-content after illumination and surface iodide-content in pristine film. Data were acquired using TOF-SIMS for $(FA_{1-x}MA_x)Pb(I_{1-y}Br_y)_3$ perovskite films with different compositions. **a** {x/y} = 0.75¦0.50. **b** {x/y} = 0.25|0.50.

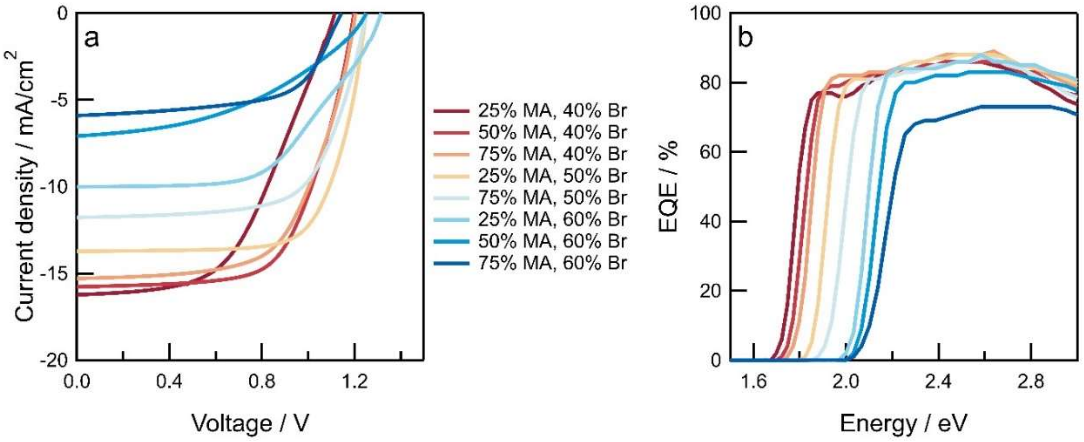

Supplementary Fig. 24 | Characterization of $(FA_{1-x}MA_x)Pb(I_{1-y}Br_y)_3$ perovskite solar cells for different compositions. a Current density versus voltage curves of solar cells used for photocurrent spectroscopy measurements. **b** Corresponding external quantum efficiency spectra.

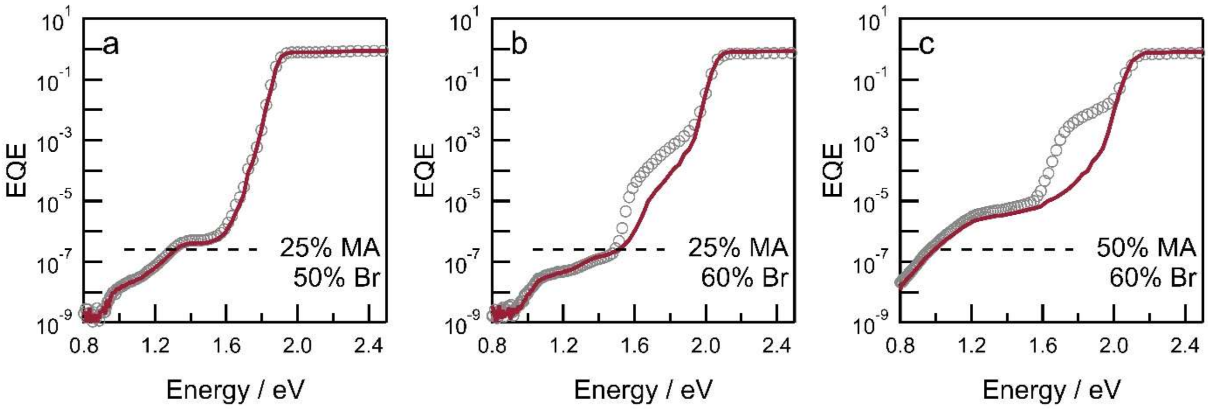

Supplementary Fig. 25 | Sensitive EQE spectra of $(FA_{1-x}MA_x)Pb(I_{1-y}Br_y)_3$ perovskite solar cells with different compositions. a {*x*/*y*} = 0.25|0.50. **b** {*x*/*y*} = 0.25|0.60. **c** {*x*/*y*} = 0.50|0.60. Dashed horizontal lines represent the sub-bandgap EQE intensity acquired for solar cell with composition {*x*/*y*} = 0.25|0.40. Solid lines represent spectra for pristine solar cells and open circles represent spectra acquired after continuous illumination.
