# Supplementary material for: Local halide heterogeneity drives surface wrinkling in mixed-halide wide-bandgap perovskites
Source: Nat Commun. 2025 Feb 25;16:1967. doi: 10.1038/s41467-025-57010-6 (PMC11861982; doi:10.1038/s41467-025-57010-6)
Supplement: Supplementary file 2 — Reporting Summary [file 41467_2025_57010_MOESM2_ESM.pdf]

Corresponding author(s): Kunal Datta & Rene Janssen

Solar Cells Reporting Summary

Nature Research wishes to improve the reproducibility of the work that we publish. This form is intended for publication with all accepted papers reporting the characterization of photovoltaic devices and provides structure for consistency and transparency in reporting. Some list items might not apply to an individual manuscript, but all fields must be completed for clarity.

For further information on Nature Research policies, including our data availability policy, see Authors & Referees.

Experimental design

Please check: are the following details reported in the manuscript?

1. Dimensions

Item	Yes	No	Details
Area of the tested solar cells	☒	☐	Methods: Sample Preparation
Method used to determine the device area	☒	☐	Methods: Sample Preparation

2. Current-voltage characterization

Item	Yes	No	Details
Current density-voltage (J-V) plots in both forward and backward direction	☐	☒	Supplementary Fig. 20a shows scans in backward direction
Voltage scan conditions<br>*For instance: scan direction, speed, dwell times*	☒	☐	Methods: Thin film and solar cell characterization
Test environment<br>*For instance: characterization temperature, in air or in glove box*	☒	☐	Methods: Thin film and solar cell characterization
Protocol for preconditioning of the device before its characterization	☒	☐	No preconditioning was applied. See Methods: Thin film and solar cell characterization
Stability of the J-V characteristic<br>*Verified with time evolution of the maximum power point or with the photocurrent at maximum power point; see ref. 7 for details.*	☐	☒	The changes in EQE after illumination are shown in Supplementary Fig. 20

3. Hysteresis or any other unusual behaviour

Item	Yes	No	Details
Description of the unusual behaviour observed during the characterization	☐	☒	Light-induced halide segregation is discussed in the paper. The effects on PV efficiency were not studied. The effect on the EQE are described.
Related experimental data	☒	☐	Supplementary Fig. 21

4. Efficiency

Item	Yes	No	Details
External quantum efficiency (EQE) or incident photons to current efficiency (IPCE)	☒	☐	Supplementary Fig. 20b
A comparison between the integrated response under the standard reference spectrum and the response measure under the simulator	☐	☒	PV efficiencies are not reported in this paper.
For tandem solar cells, the bias illumination and bias voltage used for each subcell	☐	☒	No tandem cells were studied.

5. Calibration

Item	Yes	No	Details
Light source and reference cell or sensor used for the characterization	☐	☒	PV efficiencies are not reported in this paper.
Confirmation that the reference cell was calibrated and certified	☐	☒	PV efficiencies are not reported in this paper.

Calculation of spectral mismatch between the reference cell and the devices under test

☐ Yes
☒ No

PV efficiencies are not reported in this paper.

6. Mask/aperture

Size of the mask/aperture used during testing

☒ Yes
☐ No

Shadow masks of 0.0676 were used. See Methods: Thin film and solar cell characterization

Variation of the measured short-circuit current density with the mask/aperture area

☐ Yes
☒ No

Not studied

7. Performance certification

Identity of the independent certification laboratory that confirmed the photovoltaic performance

☐ Yes
☒ No

PV efficiencies are not reported in this paper.

A copy of any certificate(s)
Provide in Supplementary Information

☐ Yes
☒ No

Not applicable

8. Statistics

Number of solar cells tested

☐ Yes
☒ No

PV efficiencies are not reported in this paper.

Statistical analysis of the device performance

☐ Yes
☒ No

PV efficiencies are not reported in this paper.

9. Long-term stability analysis

Type of analysis, bias conditions and environmental conditions
For instance: illumination type, temperature, atmosphere humidity, encapsulation method, preconditioning temperature

☐ Yes
☒ No

PV efficiencies are not reported in this paper.
